# Supplementary material for: Progerin-induced nuclear envelope remodeling is shaped by cell division and NUP153
Source: J Cell Sci. 2026 Jun 19;139(12):jcs264678. doi: 10.1242/jcs.264678 (PMC13327547; doi:10.1242/jcs.264678)
Supplement: Supplementary file 1 [file joces-139-264678_review_history.pdf]

Journal of Cell Science

Progerin-induced nuclear envelope remodeling is shaped by cell division and NUP153

Ayse Mubine Turkmen, Jing Wang, Adam Frost and Katharine S. Ullman
DOI: 10.1242/jcs.264678

Editor: Abigail Buchwalter

Review timeline
Original submission: 22 December 2025
Editorial decision: 2 February 2026
First revision received: 4 May 2026
Accepted: 19 May 2026

Original submission

First decision letter

MS ID#: jcs.264678

MS TITLE: Progerin-Induced Nuclear Envelope Remodeling is Shaped by Cell Division and NUP153

AUTHORS: Ayse Mubine Turkmen; Jing Wang; Adam Frost; Katharine S Ullman
ARTICLE TYPE: Short Report

Dear Dr Ullman,

We have now reached a decision on the above manuscript. The reviewers are overall supportive but request a few control experiments and clarifications that I anticipate will strengthen the manuscript. If you think that you can deal satisfactorily with the criticisms in revision, I would be pleased to see a revised manuscript. We would then return it to the reviewers.

Reviewer 1

SUMMARY OF THE ADVANCE MADE IN THIS PAPER AND ITS POTENTIAL SIGNIFICANCE TO THE FIELD

Abnormal nuclear morphology is a common feature of diseases involving the nuclear lamina, but the underlying mechanisms remain unclear. This study investigated how abnormal nuclear shapes arise in cells expressing progerin, the mutant protein responsible for Hutchinson Gilford Progeria Syndrome (HGPS). Using a doxycycline inducible system, the authors expressed GFP tagged progerin in U2OS cells and examined nuclear shape and morphology with advanced light and electron microscopy. Progerin accumulated in focal regions of the inner nuclear membrane (INM) in interphase-arrested cells. Associated with these foci, membrane-invaginations and multilayered structures enriched with specific INM proteins and NUP153 were identified. Notably, interphase-arrested cells did not display the characteristic lobulated or convoluted nuclei observed in HGPS cells. Instead, pronounced nuclear abnormalities emerged only after cell division. Depletion of NUP153 reduced progerin-containing foci, suggesting a role for NUP153 in their formation or maintenance. The authors propose that expansion of the INM contributes to increased nuclear surface area, leading to nuclear lobulation post mitosis. While the study highlights cell division as a key factor in abnormal nuclear shape formation, it does not establish a direct link between foci

© 2026. Published by The Company of Biologists under the terms of the Creative Commons Attribution License (https://creativecommons.org/licenses/by/4.0/).

formation and nuclear deformation. Additional control experiments would further strengthen the conclusions.

Major comments

[Please request additional experiments only if they are essential for supporting the conclusions; authors should be encouraged to highlight any claims that are preliminary or speculative, or to discuss any pitfalls or alternative interpretations in a 'Limitations' section]

1. Depleting NUP153 with siRNA significantly reduced GFP positive NE foci (Fig. 4). Did this also reduce post mitotic nuclear shape abnormalities?
2. The farnesylated tail domain of lamin B2 strikingly mimicked progerin's effects. Because both proteins are farnesylated and GFP tagged at the N terminus, control experiments—such as transient expression of untagged progerin detected with a progerin-specific antibody—are needed to rule out potential effects of the GFP tag.
3. The biological effects of progerin in arrested and released cells are markedly different (Fig. 1B-E). Are progerin levels comparable in arrested and released cells? Also, how do they compare with HGPS fibroblasts? A Western blot should be included that compares progerin expression in HGPS cells versus arrested and released U2OS cells, ideally normalized to lamin C (all detected with a lamin A/C antibody). The Western blot shown in Fig. S3 appears to be from HeLa cells, which show variable progerin expression (Fig. S3B-D).

Minor comments

1. Lamin B1 levels appear reduced only in cells expressing progerin (Fig. 1B) or the lamin B2 tail protein (Fig. 1F). Was this a consistent finding? Similar findings were observed in HGPS cells (Scaffidi & Misteli, 2005. Nat Med). The authors may want to comment whether the loss of lamin B1 contributes to foci formation or simply reflect a compensatory response to excess farnesylated protein at the INM.
2. The microscopy images in Fig. S3B-F are very dim. Please increase the brightness.
3. Please include the numbers of technical replicates and total numbers of cells analyzed in Fig. 4D-F.

First revision

Author response to reviewers' comments

Thank you for the constructive manuscript review. We are pleased that a reviewer noted this as “a thoughtful and carefully executed study” with “very elegant CLEM and EM tomography approaches.” Addressing the reviewers' suggestions has further strengthened this manuscript. Our responses are detailed below.

Reviewer 1:

Major comments

1. Depleting NUP153 with siRNA significantly reduced GFP positive NE foci (Fig. 4). Did this also reduce post mitotic nuclear shape abnormalities?

> We performed additional experiments to see if this could be addressed, but the depletion of Nup153 itself creates post mitotic phenotypes (LaJoie et al., 2022) that confound the interpretation.

2. The farnesylated tail domain of lamin B2 strikingly mimicked progerin's effects. Because both proteins are farnesylated and GFP tagged at the N terminus, control experiments—such as transient

© 2026. Published by The Company of Biologists under the terms of the Creative Commons Attribution License (https://creativecommons.org/licenses/by/4.0/).

expression of untagged progerin detected with a progerin-specific antibody—are needed to rule out potential effects of the GFP tag.

> To determine whether the GFP tag contributed to membrane-deforming effects attributed to progerin, we expressed an untagged progerin construct using the same inducible system. Cells arrested in interphase maintained overall nuclear shape, and a subset displayed pronounced focal accumulation of emerin at numerous nuclear foci, recapitulating the interphase phenotype observed with GFP-progerin (new Fig. S3E-H). Upon release from interphase arrest, these cells displayed an increase in dysmorphic nuclei, accompanied by a reduction in cells exhibiting multiple emerin-positive NE foci. This alignment of nuclear phenotypes following expression of either GFP-progerin or untagged progerin rules out potential effects of the GFP tag itself.

3. The biological effects of progerin in arrested and released cells are markedly different (Fig. 1B-E). Are progerin levels comparable in arrested and released cells? Also, how do they compare with HGPS fibroblasts? A Western blot should be included that compares progerin expression in HGPS cells versus arrested and released U2OS cells, ideally normalized to lamin C (all detected with a lamin A/C antibody). The Western blot shown in Fig. S3 appears to be from HeLa cells, which show variable progerin expression (Fig. S3B-D).

> Analysis of our GFP-progerin-expressing U2OS cells line, in which the majority of cells show detectable GFP following doxycycline induction, revealed similar expression of progerin in released versus arrested conditions in a western blot (new Fig. S3A-B, 6h induction). Moreover, we also found that untagged progerin elicited the same distinct phenotypes in arrested and released cells without changes in levels (new Fig S3E-H). To directly test whether increased progerin levels could influence nuclear morphology, we elevated progerin expression in arrested cells by extending the induction period. Although higher expression caused nuclear contour deformation in a minority of cells, nuclei overall retained an ovoid shape and the dominant phenotype remained nuclear foci (new Fig. S3C-D).
>
> Quantification of progerin, lamin A, and lamin C band intensities in arrested conditions across three independent experiments yielded an average progerin-to-lamin A ratio of 2.0 ± 0.55 and a progerin-to-lamin C ratio of 2.8 ± 1.0 in our GFP-progerin line and a progerin-to-lamin A ratio of 1.0 ± 0.1 and a progerin-to-lamin C ratio of 0.9 ± 0.2 in our untagged progerin line. Previous studies of cultured HGPS fibroblasts report progerin-to-lamin A ratios ranging from approximately 0.9 to 1.5 (Moulson et al., 2007; Gabriel et al., 2015; Dzianisava et al., 2025). Importantly, reported progerin abundance varies substantially depending on age, tissue type, and experimental system. For example, a study using an HGPS mouse model showed a wide range of relative progerin expression, with progerin-to-lamin C ratios near 1 in heart and kidney tissue and more than 5 in the aorta (Kim et al., 2018). Thus, the progerin levels observed in our assay are comparable to values reported in established human and animal models. We have now added the quantification of progerin-to-lamin A ratios (new Fig S3B, F) and context from the literature (p. 5).

<u>Minor comments</u>

1. Lamin B1 levels appear reduced only in cells expressing progerin (Fig. 1B) or the lamin B2 tail protein (Fig. 1F). Was this a consistent finding? Similar findings were observed in HGPS cells (Scaffidi & Misteli, 2005. Nat Med). The authors may want to comment whether the loss of lamin B1 contributes to foci formation or simply reflect a compensatory response to excess farnesylated protein at the INM.

> Although we occasionally observed reduced lamin B1 levels in cells expressing GFP-progerin, as reported by Scaffidi & Misteli, this was not a consistent or robust finding across experiments and is likely sensitive to additional experimental variables. While we do not find strong evidence that lamin B1 loss is a necessary prerequisite for NE foci formation, reduced lamin levels, either through global lamina thinning or localized lamina disassembly, could, in principle, facilitate INM invagination. Localized lamina remodeling or

© 2026. Published by The Company of Biologists under the terms of the Creative Commons Attribution License (https://creativecommons.org/licenses/by/4.0/).

disassembly is a well-established feature of INM deformation in diverse contexts, including nuclear lipid droplet biogenesis and viral nuclear egress (McPhee et al., 2024). While our current data do not allow us to distinguish whether lamin B1 reduction plays a causal role in foci formation or instead reflects a secondary, compensatory response to excess farnesylated protein at the INM, we think this would be interesting to test in the future.

We have added additional western blots showing lamin B1 levels (new Fig. S3B, F) and the related findings in the text (p.5, line 25-27).

2. The microscopy images in Fig. S3B-F are very dim. Please increase the brightness.

We have adjusted all images in what is now Fig. S4 in a uniform and linear manner.

3. Please include the numbers of technical replicates and total numbers of cells analyzed in Fig. 4D-F.

The same cells were analyzed in 4D-F. We have made this clearer (p. 21).

Reviewer 2:

<u>Minor comments</u>

1. Could the authors please estimate, or comment on, how the doxycycline-induced level of progerin expression compares to the levels of progerin has it been expressed from its endogenous promoter (perhaps based on Figure S3A)?

See comment #2 above.

2. It would be beneficial to the reader if the authors speculated on the reason for formation of foci when are farnesylated nuclear proteins are expressed in interphase, either using the “lamina stability” explanation provided above, or another explanation that the authors may find more fitting.

We appreciate the reviewer’s comments and have incorporated a model in the text (p. 5-6) in which progerin, due to its inability to integrate into the pre-existing interphase lamina, potentially reflecting the intrinsic stability of the lamina and/or properties unique to progerin, assembles instead into an alternative lamina at discrete permissive sites. These sites may correspond to regions where the lamina is weakened and/or where coupling between the inner and outer nuclear membranes is reduced, thereby allowing localized detachment and invagination of the INM. Extending this model, passage through mitosis may permit assimilation of progerin into the lamina network and, along with it membrane that otherwise would pack within INM invaginations.

3. On Page 4, line 34: the authors state that “To test whether the two stages of NE phenotype described above (Fig 1B-E) are specific to progerin or represent a broader effect of excess farnesylated lamins….”. Formally, the authors did not exclude the possibility that any lamin, even if it isn’t constitutively farnesylated, would form foci when over expressed in interphase. If this is known to not be the case, it should be noted (and cited). Otherwise, this possibility should be acknowledged.

The highly folded nuclear membranes observed by Prüfert et al., 2004 upon expression of lamins containing a farnesylated CaaX motif were not observed when lamin C, which lacks this motif, was expressed, nor when the farnesylated cysteine of lamin B2 was mutated to serine, thereby preventing farnesylation. We have added this comparison to the text (p. 4) to clarify that focal nuclear envelope deformations are not a general consequence of lamin overexpression, but rather are specific to lamins capable of undergoing farnesylation.

4. Regarding the use of Emerin and Nesprin2 to visualize the inner and outer nuclear membranes (top of page 5): For the non-aficionado reader, it might be helpful to note that the INM and OMN

© 2026. Published by The Company of Biologists under the terms of the Creative Commons Attribution License (https://creativecommons.org/licenses/by/4.0/).

are too close to be distinguished by light microscopy, but this approach is useful to determine whether only the inner or outer membranes form protrusions or invaginations.

Thank you for this suggestion – added (see p. 4)

5. Page 5, line 30: the authors state that "…Then, rather than reforming with this topology after division, excess membrane material contributes to the increased nuclear surface area and emergence of the lobulated nuclear morphology characteristic of HGPS." This makes it sound as if the membrane is the driving force behind INM expansion into whorls etc. Isn't a more likely explanation that after nuclear division, the over expressed progerin no longer form foci (possibly because it is incorporated into the newly formed lamina) and therefore the INM no longer forms intranuclear extensions?

As stated in response to this reviewer's second comment, we now include this explanation in our discussion (p. 5-6)

6. There are some minor inconsistencies in Figure 1A: The authors state (page 3, line 31) that "GFP-progerin localized to the nuclear rim approximately 4 hours post doxycycline induction", but the image is labeled t=5. Also, in the legend they state that "Images were acquired every 30 minutes for 5 hours", but the Figure shows an image from t=7. Either the imaging didn't start at t=0, or they were acquired for at least 7 hours.

Thank you for pointing this out --we have corrected the labels and the text.

Second decision letter

MS ID#: jcs.264678R1

MS Title: Progerin-Induced Nuclear Envelope Remodeling is Shaped by Cell Division and NUP153

Authors: Ayse Mubine Turkmen; Jing Wang; Adam Frost; Katharine S Ullman
Article Type: Short Report

Dear Dr Ullman,

I am happy to tell you that your manuscript has been accepted for publication in Journal of Cell Science, pending standard publication integrity checks.

© 2026. Published by The Company of Biologists under the terms of the Creative Commons Attribution License (https://creativecommons.org/licenses/by/4.0/).
